# The Occurrence of the Cicada *Cicadatra persica* on Apple Trees, *Malus domestica,* in Erneh, Syria

**DOI:** 10.1673/031.013.4201

**Published:** 2013-05-21

**Authors:** Marah A. Dardar, Hamzeh M.R. Belal, Abedlnabi M. Basheer

**Affiliations:** 1 General Commission for Scientific Agricultural research, Administration of Plant Protection Research, Dep. Insect Research, Douma, Damascus, Syria; 2 University of Damascus, Faculty of Agriculture, Dep. Plant Protection, Damascus, Syria; 3 University of Damascus, Faculty of Agriculture, Dep. Plant Protection, Damascus, Syria

**Keywords:** acoustic analysis, apple orchards, Cicadidae, morphological characters

## Abstract

An infestation of *Cicadatra persica* KirKaldy (Hemiptera: Cicadidae) on apple trees, *Malus domestica* Borkhausen (Rosales: Rosaceae), was reported for the first time in the apple fruit orchards of Erneh, Syria. Nymphs, adults, exuvia, and exit holes in the soil were observed. The species was identified as *C. persica* based on morphological characters. Some biological observations and an acoustic analysis of the male's songs were also achieved.

## Introduction

*Cicadatra persica* Kirkaldy (Hemiptera: Cicadidae) was previously reported in Iran, the Caucasus, Asia Minor, and the Mediterranean island Sicily, and its sound signals were described for the first time in Macedonia (Gogala and Trilar 1998). Demir (2008) also reported *C. persica* in Monaco, Sicily, the Near East, and Turkey. Ahmed and Sanborn (2010) also reported it in Pakistan.

### Observation of *Cicadatra persica*

An infestation of *C. persica* was observed in June 2011 causing severe damage to *Malus domestica* Borkhausen (Rosales: Rosaceae) trees in the village of Erneh, which is located on Al-sheikh Mountain in the southwest of Syria. Nymphs of this insect were observed on the roots, while adults and exuvia were observed on the grass and ground surrounding the trees, as well as on the trunks of the trees. Exit holes of nymphs were observed in the soil around the trees. The damage done by this species is caused by the nymphs, which attack *M. domestica* roots under the ground, and by the adults, which lay their eggs inside the branches, leading to a fall of leaves and a reduction of growth. The species was identified as *C. persica,* according to its morphological characters. Some biological observations and an acoustic analysis of the male's songs were also made.

### Collecting individuals

Adults were manually collected from branches of *M. domestica* at sunrise and sunset because they are easily collected at these times. They were collected with an emergence trap which is a tent-like structure made of a cloth-screen (2 m diameter) around *M. domestica* trees. Nymphs able to move were collected by digging 10–15 cm deep in the soil.

### Morphological characters of *C. persica*

**Figure 1.**
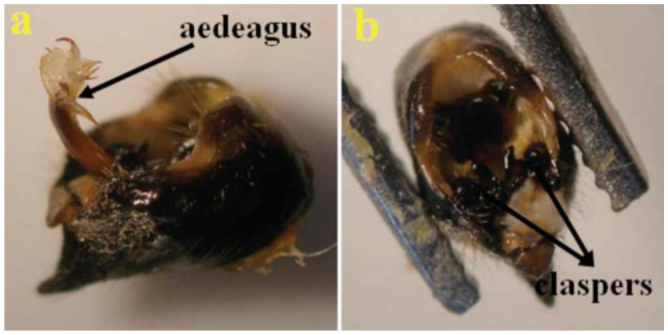
Aedeagus (a) and claspers (b) of male genitalia of *Cicadatra persica.* High quality figures are available online.

**Figure 2.**
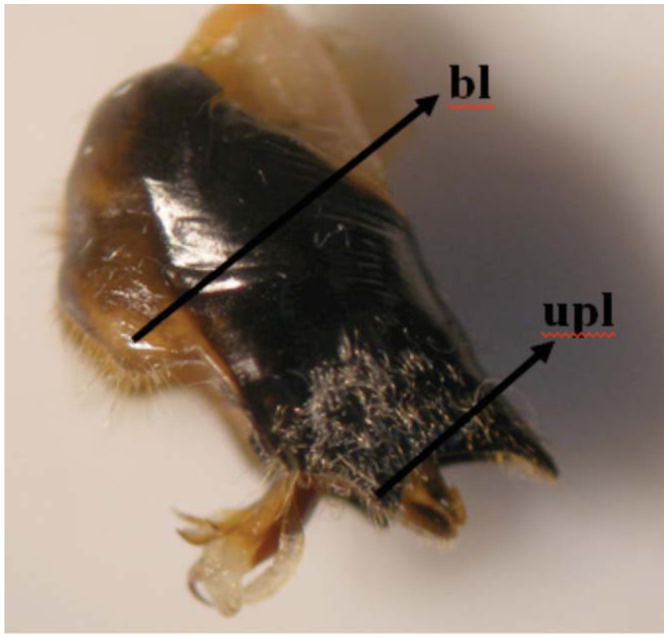
Pygofer of *Cicadatra persica.* b1: based lop of Pyrofer. up1: the external upper lop of pygofer. High quality figures are available online.

**Figure 3.**
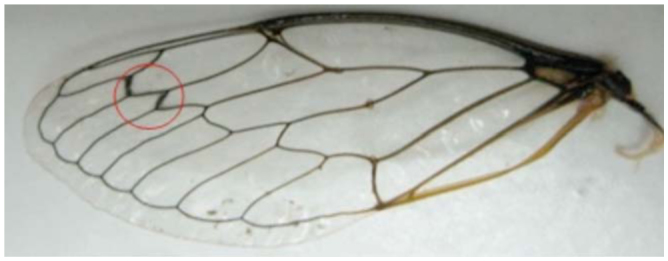
Exterior edge of U1 and U2 cells of forewings or *Cicadatra persica.* High quality figures are available online.

Adults have fewer light patterns on the dorsal body surface, and have a blackish appearance. There is only one median yellow mark posterior to the ocelli. The supra-antennal plate is dominantly yellow. The pronotum is without a narrow yellow edge band on the posterior edge. The male opercula do not reach the anterior margin of sternite II. The vein between cells U1 and U2 and the apical vein of the forewing radial cell are curved. The claspers bear golden, long piles and are not sickle-like. The uncus is long, extending towards the stem of the aedeagus. A long membranous part is present in Figure 1a, b. The external upper lop of the pygofer is shown in Figure 2. The exterior edges of the U1 and U2 cells of the forewings are distinctive (Figure 3).

### Acoustic analysis

Sounds of 3 ♂♂ of cicadas were recorded in the orchards of Erneh at a temperature between 28° and 3O°C. The sounds were then analyzed by Speech Analyzer 3.0.1 program (SIL International, www.sil.org). The film of the male's songs was recorded by using a camera (Canon Power Shot A3100 *IS*, 4× optical zoom, 12.1 mega pixels, www.canon.com).

### The courtship song

**Figure 4.**
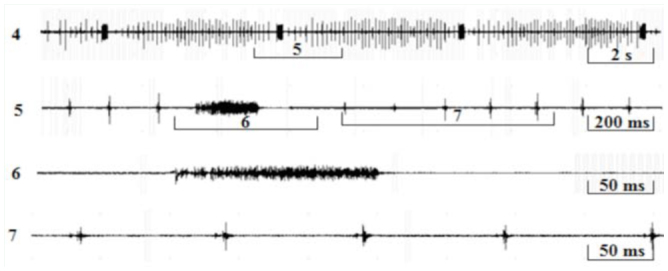
Oscillograms of the calling sounds with wings clicking of male *Cicadatra persica.* High quality figures are available online.

**Figure 5.**
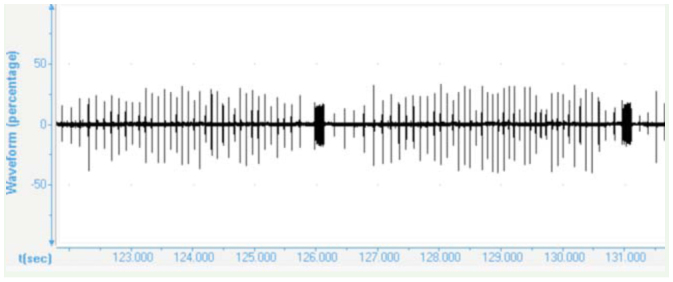
Oscillograms of the calling sounds with wings clicking of male *Cicadatra persica.* High quality figures are available online.

**Figure 6.**
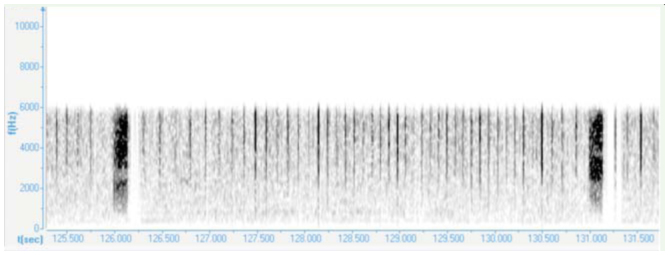
Spectrogram analysis of the courtship song of *Cicadatra persica.* High quality figures are available online.

The phrases of the courtship song consisted of a long series of rapidly repeated wing clicks (mean repetition period: 75.7 ± 0.7 ms) ending with a loud tymbal echeme with increasing amplitude (duration: 161 ± 3.04 ms; Figure 4, 5). The mean period duration of such repeating phrases was 5.12 ± 0.1 sec. After a short pause (Figure 5, 6) the wing clicks of the next phrase started again. The first interval between the last click and a tymbal echeme was extremely variable and lasted between 60 and 244 ms (mean: 149 ± 26.6 ms). The average number of wing clicks in a phrase was 46 ± 1.8 (Figure 5).

### The continuous song

**Figure 7.**
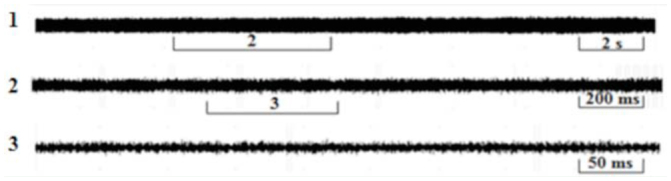
Oscillograms of the continuous sounds of male *Cicadatra persica.* High quality figures are available online.

**Figure 8.**
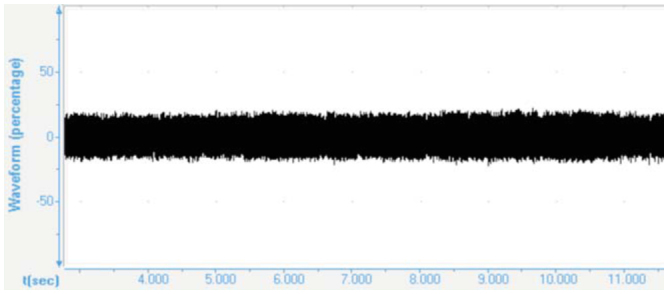
Waveform (percentage) of the continuous song of *Cicadatra persica.* High quality figures are available online.

The phrases in the continuous song lasted without interruption for many minutes (Figures 7, 8). The frequency ranged between 1051.8 Hz ± 41.96 Hz and 5745.1 Hz ± 10.49 Hz, with a maximum amplitude between 2725.8 ± 24.30 Hz and 4705.1 ± 52.92 Hz.

The general patterns of sound emissions were the same as in Gogala and Trilar's recordings (1998). However, the spectra of recordings of tymbal sounds (continuous and courtship) were quite different from Gogala and Trilar's recordings (1998).

The possible explanation could be of a technical nature. The video camera that was used a frequency range limited to 15.5 kHz, and the spectral properties of the whole spectral range of sound recordings couldn't be known. The sound in the camera was recorded as a compressed format, which may have affected the recordings.

### Biological observation

The egg-nest of *C. persica* contained a number of slits, and each slit included numbers of eggs. The median number of eggs in the slits increased or decreased depending on the length and depth of the slit. The medium number of eggs per nest varied depending on the nest length and the number of slits per nest. In this study, 60 nests (30 nests ≤ 4 cm, and 30 nests > 4 cm) of *C. persica* were examined. The results showed that the medium number of eggs per slit was 11. The correlation coefficient between the number of eggs per nest, the number of slits per nest, and the length of the egg-nest were also studied. The results showed a strong positive correlation between the number of eggs per nest, the number of slits per nest, and the length of the nest (R = 0.973 between the number of eggs per nest and the number of slits per nest, R = 0.914 between the number of slits per nest and the length of the nest, R = 0.881 between the number of eggs per nest and the length of the nest).

To determine the median number of eggs per nest, a study would be needed to make a linear regression analysis between the number of eggs per nest, the number of slits per nest, and the length of egg nest, in order to find an equation of the medium number of eggs per nest by indexing the number of slits per nest and the length of the nest.

The medium number of egg nests and eggs per female could not be determined in the same season because it was difficult to isolate a virgin female and promote its mating and egg-laying behavior. The field observation showed that the nest scars showed in the live branches, but they did not result in the death of branches.

The first observation of the adults in the orchards was on 7 June 2011, and the first observation of egg-laying was on 14 June 2011. The time between the two observations showed that the females become ready to mate and lay eggs, and the males start to sing and mate, a few days after exclusion. The pairs remain in copula for an average of 30 to 35 minutes.

The infestation of *C. persica* on *M. domestica* shows the damage potential of this species and the necessity of developing integrated management programs for species of this group. It was difficult to quantify crop loss compared to non-infested trees or orchards in this study because special research over several seasons is needed.

## References

[bibr01] Ahmed Z, Sanborn AF. (2010). The cicada fauna of Pakistan including the description of four new species (Hemiptera: Cicadoidea: Cicadidae).. *Zootaxa*.

[bibr02] Demir E. (2008). The fulgoromorpha and Cicadomorpha of Turkey. Part I: Mediterranean region (Hemiptera).. Munis Entomology and Zoology.

[bibr03] Gogala M, Trilar T. (1998). First record of *Cicadatra persica* Kirkaldy, 1909 from Macedonia, with description of its song.. *Acta Entomologica Slovenica*.

